# The Concept of Using the Decision-Robustness Function in Integrated Navigation Systems

**DOI:** 10.3390/s22166157

**Published:** 2022-08-17

**Authors:** Krzysztof Czaplewski, Bartosz Czaplewski

**Affiliations:** 1 Department of Navigation, Faculty of Navigation, Gdynia Maritime University, 81-374 Gdynia, Poland; 2 Department of Teleinformation Networks, Faculty of Electronics, Telecommunications and Informatics, Gdansk University of Technology, 80-233 Gdansk, Poland

**Keywords:** intelligent transportation systems, marine navigation, marine safety, positioning system

## Abstract

The diversity and non-uniformity of the positioning systems available in maritime navigation systems often impede the watchkeeping officer in the selection of the appropriate positioning system, in particular, in restricted basins. Thus, it is necessary to introduce a mathematical apparatus to suggest, in an automated manner, which of the available systems should be used at the given moment of a sea trip. Proper selection of the positioning system is particularly important in integrated navigation systems, in which the excess of navigation information may impede the final determinations. In this article, the authors propose the use of the decision-robustness function to assist in the process of selecting the appropriate positioning system and reduce the impact of navigation observations encumbered with large errors in self-positioning accuracy. The authors present a mathematical apparatus describing the decision function (a priori object), with the determination of decision-assistance criteria, and the robustness function (a posteriori object), with different types of attenuation function. In addition, the authors present a computer application integrating both objects in the decision-robustness function. The study was concluded by a test showing the practical application of the decision-robustness function proposed in the title.

## 1. Introduction

Researchers have been fascinated by the coexistence of humans, fauna and flora from the beginning of life on Earth. The more we learn about the relationships between them, the better we understand the deep coexistence of such different worlds. In addition, as science and technology develop, we will need to integrate and jointly use apparently independent systems. In general, a system is an assembly of mutually-coupled components that serves a specific purpose and is considered detached from the environment for a specific purpose (descriptive, research or other). Based on the general definition, each component, or subsystem it includes, works to ensure system functionality and integration. Similarly, in different navigation type areas, the integration of apparently independent systems has been visible for years, of which the joint objective is to ensure a high safety level. To detail the above definition for the purposes of navigation, it may be stated [1] that a system is “…a combination of equipment and software which use interconnected controls and displays to present a comprehensive site of navigational information to the mariner…”.

In 1996, the International Maritime Organisation adopted performance standards [2] for integrated navigation bridge systems (IBSs). The IBS is any combination of systems interconnected to enable centralised access to information or the execution of commands by system components to perform two or more of the following operations [1]: passage execution, communications, machinery control, loading, discharging and cargo control, safety and security.

Contemporary integrated navigation systems (INSs) may be defined [3,4] as complex navigation systems capable of performing at least the following tasks: collision and excessive approach prevention, ship route monitoring and, simultaneously, enabling the navigator to plan, monitor and safely navigate the ship. It should be assumed that INSs are independent navigation systems used for self-positioning and other purposes. At present, satellite systems [5] and radar systems [6,7] are predominantly used in maritime navigation.

The predominance of global navigation satellite systems (GNSSs) is due to advantages such as positioning accuracy, availability and reliability. Unfortunately, the satellite systems included in GNSSs are increasingly prone to noise; hence their reliability may be lower than expected by the users. The causes and types of noise have been described, for example, by [8,9,10]. At this point, one should note the purposeful operation of GNSS jamming and spoofing systems and equipment. Although much time has been dedicated to the methods of handling spoofing and jamming [11,12], some authors, e.g., [13,14], note the requirement to have a different positioning system as an alternative to the GNSS, which may be essential for navigation and object positioning assistance.

On the other hand, radar systems, by definition, have a lower positioning accuracy; however, they are a very good alternative to satellite systems in confined basins. However, it was generally believed until recently that radar-based positioning has low accuracy and is relatively labour intensive. Contemporary technology and the development of data-processing methods, combined with modern IT systems, enable a significant increase in positioning accuracy, as well as reliability and rate.

In this article, the authors present the results of another stage of their research on the adaptation, for the purposes of maritime navigation, of the method of robust estimation proposed in [15], which uses bi-factor equivalent weights to equalise geodetic observations. The result of this research is the proposition to use automated decision-robustness functions in the systems, aimed at identifying the “useful” positioning systems, from the perspective of international resolutions, and reducing the impact of observations, encumbered with large errors, on the final self-positioning of the ship at sea. The article presents the robust estimation methods applicable to maritime navigation and the proposition to automate the selection of the navigation system to use for ship positioning. The entirety was subject to theoretical tests, of which one is presented in this article.

The authors’ contribution to the literature is:

- a new concept of using the decision-robustness function in maritime navigation;
- a new technique for selecting the best navigation system based on the concept of the decision-robustness function;
- new possibilities of using the function in one-man-bridge systems or in autonomous vehicle algorithms;
- increasing the range of available methods in the automation of navigation processes thanks to the new concept of the decision function.

The decision-robustness function was introduced to the literature for the first time in [16]. Novelty in this article is:

- the adaptation of the decision-robustness function to the needs of maritime navigation;
- the possibility of using the decision-robustness function to solve typical navigation tasks.

The structure of the article is as follows. Section 2 describes the related work. A mathematical description of the proposed model is included in Section 3. Section 4 contains the results of the experiments. Conclusions are included in Section 5.

## 2. Related Work

No INS component will operate properly without the algorithms that integrate and utilise the output of INS components. Attempts at integrating the navigation information from different positioning systems have been undertaken in the past [17,18]. The work [17] addresses the issue of safety in areas with very high traffic of ships at sea. This paper discusses a solution that integrates data from coastal radar stations, AIS and vision systems in order to determine the position of ships. A degree of error is discussed which depends on the specific system used. In order to increase the precision of position fixing, the authors of [17] proposed two geodesic methods of estimation and one innovative method for VTS systems.

Our proposed method has its origin in [16]. This publication is the result of the habilitation work of the main author and contains the theoretical foundations of the solution developed and proposed in this paper. In [16] one can find details on positioning with interactive navigational structures implementation.

Other research centres have also attempted to implement known mathematical methods for the joint use of navigation information from different navigation systems. For example, refs. [19,20] presented the application of the adaptive interacting multiple model (AIMM) filter method and the expanded Kalman filter (EKF) for the integration of navigation information from GNSSs and INSs. The authors of [19,20] proposed new methods to avoid the weaknesses of EKF, i.e., instability and significant computational requirements. One of the most important advantages of [19] is that this solution was verified on a real ship.

There are no solutions in the literature similar to the method described in this paper. In particular, the decision function is innovative. Thus, there are no publications in the literature for direct comparison with the method proposed in this paper. Therefore, the last few related works described in this section focuses on the description of complementary methods, rather than competing, methods.

The authors of [21] raised the problem of ship traffic in ports in the presence of dangerous cargo. The method described in [21] is an analytical and methodological scheme for calculating the risk. It was stated that port regulations arose out of general experience of experts and not from quantitative methods. The aim was to show that the analytical method of risk determination can help in defining port policy regulations. The authors of [21] presented a multi-stage methodology based on expert study and real-time simulations, the result of which is the characteristics of the ship’s safe approach, i.e., parameters such as velocity, approaching angle and more. The method proposed in this paper can cooperate with the method presented in [21] in two ways. On the one hand, our proposed method may provide a decision on the best navigation system at a given moment, which will be one of the input data for the method from [21]. On the other hand, the output parameters of the method described in [21] could influence the more advanced forms of the decision function.

The work published in [22] is similar to our proposed method in that it also describes an automated method to solve the problem of the precision of the geographic coordinates of a vessel at sea. In [22] the problem of precision of measured radar distance to the edge of the echo is raised in cases where there are multiple radar echoes, or the observed vessel is so large that the edge of the echo cannot be considered as the vessel’s position. An analytical method for correcting radar distance has been introduced, assuming that the radar echo has an elliptical shape. The results of [22] support the process of determining the ship position in VTS systems with increased accuracy. Although the purpose of [22] is aligned with the purpose of our article, work [22] focuses on only one type of navigation system, namely radars, and the idea of our proposed method is to select the best system from a collection of multiple navigation systems. On the other hand, effective determining radar distances significantly affect the quality of determining the observation position. In this regard, conclusions drawn from [22] may have an impact on the future forms of the decision function.

To ensure the continuity and reliability of the navigation process, many alternative navigation systems have been examined in recent years, including vision, radar, laser, ultrasound, ultra-wide band (UWB) and eLoran sensors [23,24]. In coastal areas and areas featuring multiple landmarks (radar and navigation marks), systems using vision or radar sensors have become particularly important [25,26]. The use of camera images, e.g., mounted on unmanned aerial vehicles (UAV), is particularly interesting. For example, the authors of [27] proposed a method to correct spatial orientation angle based on a shoreline image for use in coastal and port environments. The use of this type of tool, such as passive cameras, develops the interdisciplinary aspect of navigation, reaching to the achievements of image processing, signal processing and robotics. Nowadays, image-based navigation should be seen as another potential source of data for position-fixing algorithms. Although these solutions are not directly included in this article, since we focus on GNSS and radar in the following chapters, the set of navigation systems supported by our proposed method could be extended to include visual or laser systems in the future.

## 3. Description of Mathematical Model

### 3.1. Robust Estimation Task and Its Solution

In measurement practice, one sometimes encounters situations in which measurement results are encumbered with large errors. In general, these errors result from regular misreading of navigation equipment indications, misidentification of the observed navigation marks or a change in hydro-meteorological conditions during the acquisition of measurement data. In such situations, geodetic robust estimation methods may be used, known and well described in the source literature [28,29]. At present, as many navigation systems are available, their diversity may impede safe navigation instead of supporting it. On the other hand, joint use of the navigation data from different navigation positioning system types should be considered. For this, we assumed access to navigation information from differential GPS (DGPS) and radar measurements, and we assumed bi-dimensional ship positioning $\left(X_{P},Y_{P}\right)$. Furthermore, the position coordinates were included in the vector $X={\left[X_{P},Y_{P}\right]}^{T}$. In addition, following the generally accepted formation principles of observation equation systems [30], we combined observations from two independent positioning systems in one observation equation system:

(1) $$\left.\begin{array}{l} y_{R_{1}}+v_{R_{1}}=F_{R_{1}}\left(X_{1},X_{2},\ldots ,X_{r}\right)\\ \vdots \\ y_{R_{i}}+v_{R_{i}}=F_{R_{i}}\left(X_{1},X_{2},\ldots ,X_{r}\right)\\ y_{D_{1}}+v_{D_{1}}=F_{D_{1}}\left(X_{1},X_{2},\ldots ,X_{r}\right)\\ \vdots \\ y_{D_{j}}+v_{D_{j}}=F_{D_{j}}\left(X_{1},X_{2},\ldots ,X_{r}\right) \end{array}\right\}\;\Leftrightarrow y+v=F\left(X\right)$$

where $y_{R}={\left[y_{R_{1}},y_{R_{2}},\ldots ,y_{R_{n}}\right]}^{T}$ is the radar observation vector, while $y_{D}={\left[y_{D_{1}},y_{D_{2}},\ldots ,y_{D_{n}}\right]}^{T}$ is the DGPS observation vector, with combined results in the vector $y={\left[y_{R},y_{D}\right]}^{T}$. $v_{R}={\left[v_{R_{1}},v_{R_{2}},\ldots ,v_{R_{n}}\right]}^{T}$ is understood as the radar observation correction vector, while $v_{D}={\left[v_{D_{1}},v_{D_{2}},\ldots ,v_{D_{n}}\right]}^{T}$ is understood as the DGPS observation correction vector which, combined, enables the formation of the vector $v={\left[v_{R},v_{D}\right]}^{T}$. Vector v is a vector of random observation errors with the covariance matrix, $C_{v}$, and the expected value vector, E(v)=0. If it is assumed that the antenna of the radar and GNSS receiver (in this case: DGPS) is placed on the ship in the same position, with known approximate coordinates, then one may form the vector $X^{0}={\left[X_{P}^{0},Y_{P}^{0}\right]}^{T}$ and replace the vector $y+v=F\left(X\right)$ with the linear observation equation in the following form:

(2) $$v=F\left(X\right)-y=F\left(X^{0}+d_{X}\right)-y={\left.\frac{\partial F\left(X\right)}{\partial X}\right|}_{X=X^{0}}d_{X}+F\left(X^{0}\right)-y=Ad_{X}+l$$

The value $d_{X}$ is the vector of unknown coordinate increases, so that $X=X^{0}+d_{X}$. The matrix:

(3) $$A={\left.\frac{\partial F\left(X\right)}{\partial X}\right|}_{X=X^{0}}={\left[\begin{array}{cc} \frac{\partial F_{R_{1}}\left(X\right)}{\partial X_{P}} & \frac{\partial F_{R_{1}}\left(X\right)}{\partial Y_{P}}\\ \cdots & \cdots \\ \frac{\partial F_{R_{i}}\left(X\right)}{\partial X_{P}} & \frac{\partial F_{R_{i}}\left(X\right)}{\partial Y_{P}}\\ \frac{\partial F_{D_{1}}\left(X\right)}{\partial X_{P}} & \frac{\partial F_{D_{1}}\left(X\right)}{\partial Y_{P}}\\ \cdots & \cdots \\ \frac{\partial F_{D_{j}}\left(X\right)}{\partial X_{P}} & \frac{\partial F_{D_{j}}\left(X\right)}{\partial Y_{P}} \end{array}\right]}_{X=X^{0}}$$

is a known matrix of factors, while

(4) $$l=F\left(X^{0}\right)-y$$

is the vector of free expressions. Considering the above, one may form the estimation task known in geodesy [30] as:

(5) $$\left\{\begin{array}{l} v=Ad_{X}+l\\ C_{y}=\sigma_{0}^{2}Q_{y}=\sigma_{0}^{2}P_{y}^{-1}\\ \underset{d_{X}}{\min}\left\{\xi \left(d_{X}\right)=v^{T}P_{y}v\right\}=v^{T}P_{y}v \end{array}\right.$$

where $v=\left[\begin{array}{l} v_{R}\\ v_{D} \end{array}\right]$ is the completed observation corrections vector, $C_{y}$ is the observation results covariance matrix, $Q_{y}$ is the known co-factors matrix, $\sigma_{0}^{2}$ is the unknown variance factor and $P_{y}=Diag\left(P_{R},P_{D}\right)$ is the completed observations weight matrix.

The classic form of the estimation task enables the following solution to be presented: $d_{X}=-{\left(A^{T}P_{y}A\right)}^{-1}A^{T}P_{y}l$; the final estimation of the ship position coordinates may be obtained from the following relationship: $X=X^{0}+d_{X}$. However, this approach considers the situation in which we use observations that are not encumbered with large errors or that are compliant with the requirements of international organisations related to the selection of navigation system types in a specific sea basin. In other cases, it is proposed to use the decision-attenuation function, which is a particular bi-factor of the equivalent weight matrix described in [15]. The theoretical basis for the formation and application of the decision-robustness function is described in [16]. In the case of practical navigation problems, it is possible to specify, even before the estimation (a priori), those components of the set of observations that should have no impact on the final determination (e.g., non-compliant with formal and legal requirements or identified false radar echo, for which the observation was automated). As assumed in [16], these observations correspond to zero values in the decision function. In the robust estimation process, the replacement of the decision function with the more general attenuation function eventually leads to the desired results. However, when a priori information on unacceptable observations is available, one may propose a different, more reasonable solution in practical terms. The basis for this proposition is the combination of the decision function (a priori object) and the attenuation function (a posteriori object). This way, the decision-robustness function is obtained [16]:

(6) $$\tilde{t}\left(y,\upsilon \right)=t\left(y\right)t\left(\upsilon \right)$$

with general properties:

(7) $$\tilde{t}\left(y,\upsilon \right)=\left\{\begin{array}{ll} 0 & \mathrm{for}\;y\;\mathrm{unacceptable}\;a\;\mathrm{priori}\\ t\left(\upsilon \right) & \mathrm{for}\;y\;\mathrm{participating}\;\mathrm{in}\;\mathrm{estimation} \end{array}\right.$$

The decision-attenuation function may be the basis for the formation of the decision-robustness matrix:

(8) $$\tilde{T}(y,v)=T\left(y)T(v\right)$$

where $T\left(y\right)$ is the decision matrix based on the decision function $t\left(y\right)$ (a priori object) and $T\left(v\right)$ is the attenuation matrix based on the attenuation function $t\left(v\right)$ (a posteriori object).

Based on the decision-robustness matrix, the following equivalent weight matrix is obtained:

(9) $${\tilde{\overset{⌢}{P}}}_{y}=T\left(y)T(v\right)P_{y}T\left(y)T(v\right)=T\left(y\right)\underset{{\overset{⌢}{P}}_{y}}{\underbrace{T(v)P_{y}T(v)}}T\left(y\right)=T\left(y\right){\overset{⌢}{P}}_{y}T\left(y\right)$$

Assuming that ${\tilde{\overset{⌢}{p}}}_{i}=t\left(y,v_{i}\right)p_{i},\;i=1,\ldots ,n$, the equivalent matrix for independent navigation observations (in the considered problem, from different navigation systems) will take the following form:

(10) $${\tilde{\overset{⌢}{P}}}_{y}=\tilde{T}\left(y,v\right)P_{y}=\left[\begin{array}{cccc} {\tilde{\overset{⌢}{p}}}_{1} & & & \\ & {\tilde{\overset{⌢}{p}}}_{2} & & \\ & & \ddots & \\ & & & {\tilde{\overset{⌢}{p}}}_{n} \end{array}\right]=\left[\begin{array}{cccc} t\left(y,v_{1}\right)p_{1} & & & \\ & t\left(y,v_{2}\right)p_{2} & & \\ & & \ddots & \\ & & & t\left(y,v_{n}\right)p_{n} \end{array}\right]$$

Following the above assumptions, the final estimation task will take the following form:

(11) $$\left\{\begin{array}{l} v=Ad_{X}+l\\ {\tilde{\overset{⌢}{C}}}_{y}=\sigma_{0}^{2}{\tilde{\overset{⌢}{P}}}_{y}^{-1}\\ \underset{dX}{\min}\left\{\xi \left(dX\right)=v^{T}{\tilde{\overset{⌢}{P}}}_{y}v\right\}=v^{T}{\tilde{\overset{⌢}{P}}}_{y}v \end{array}\right.$$

The diagonal components of the correction vector covariance matrix estimator v in the form of ${\hat{C}}_{v}=m_{0}^{2}\left[P_{y}^{-1}-A{\left(A^{T}P_{y}A\right)}^{-1}A^{T}\right]$ are squares of the mean determination errors of the appropriate corrections, $v_{i}$. In accordance with [30], it turns out that due to the existence of large errors, even if weight values are selected properly, one may expect a high value of large errors or high values of the variance factor estimator, $m_{0}^{2}$. Thus, special methods of robust estimation of the variance factor should be used, as proposed in, e.g., [31,32]. The occurring overestimates of mean errors, $m_{\hat{v}}$, may disrupt the identification of outlying observations because the values of standardised correction estimators in the form of $\bar{\hat{v}}=\frac{\hat{v}}{m_{\hat{v}}}$ will be underestimated. Hence, assuming that $m_{0}^{2}=1$, the covariance matrix estimator of the correction vector will take the following form:

(12) $${\hat{C}}_{v}=P_{y}^{-1}-A{\left(A^{T}P_{y}A\right)}^{-1}A^{T}$$

In view of such assumptions, the standardised corrections occurring in attenuation functions are in practice replaced with standardised estimators in the following form:

(13) $${\bar{\hat{v}}}_{i}=\frac{{\hat{v}}_{i}}{m_{{\hat{v}}_{i}}}=\frac{{\hat{v}}_{i}}{\sqrt{{\left[{\hat{C}}_{v}\right]}_{ii}}}$$

As the equivalent weight matrix, ${\tilde{\overset{⌢}{P}}}_{y}$, depends on standardised corrections, the estimation task solution (Equation (11)) and thus the determined increases $\left(d_{X}\right)$ of the ship coordinates vector, $X^{0}$, is iterative. The first step in this process is estimation using the classic least squares method, which is estimation using the original weight matrix $\left(P_{y}\right)$. The next step is estimation using the least squares method, by application of the continuously modified weights attenuated by functions with the values calculated based on the corrections obtained in the previous step. The iteration ends in such an equation, in which the obtained correction values no longer differ significantly from the values from the previous equation. The general characteristics of the applied attenuation function and decision function are presented further in this article.

### 3.2. Decision Function as an a Priori Object

The research presented in the article refers to the proprietary concept of using the “decision-robustness function” applicable to the navigation process conducted in the coastal zone. Sea basins in coastal zones are considered as surface- or depth-restricted basins [33,34]. Restrictions in coastal zones, as well as international resolutions on navigation accuracy depending on the type of human activity conducted at sea and navigation areas, almost impose the requirement to use methods that connect to the surrounding ground navigation infrastructure (radar and navigation marks and identified dangerous objects), of which the relatively close distance to the ship will have an impact on the decision-making process in the selection of the positioning system and type of observations and measurement frequency.

As mentioned above, the coastal zone is characterised by the existence of ship navigation marks. These marks include stationary and floating navigation marks, port and coast marks, designated traffic separation zone marks, waterways, isolated dangers and basins closed for navigation. They have their intended purpose and contribute to higher safety, so it is recommended, by international regulations, to use them for navigation. The available navigation infrastructure is known to the navigator on the ship, and the detailed description is included in navigation sources, such as the Admiralty Sailing Directions [35], Admiralty List of Radio Signals and Admiralty Light and Fog Signals [36]. Contrary to the satellite systems, the position determined based on these marks is directly connected to the dangers in the basin.

In this study, we attempted to use the systems included in the GNSS and the radar system (radar on the ship and characteristic echoes around the basin). The information on the GNSS receiver positioning accuracy at a given moment is always available, whereas no such current information is available for the radar system on the ship. The accuracy of positioning based on independent radar measurements, of which the statistical errors are in a normal distribution, may be characterised using a practical measure often used in analyses, i.e., the mean error. As the mean error is the function of measurement accuracy and the so-called geometric factor, of which the value depends on the ship position relative to navigation marks (radar echoes), it is reasonable to have its distribution in the considered basin.

The mean error, as the measure of positioning accuracy, will have an impact on the operation of the decision function used at the stage of selecting the type of navigation system for ship positioning. It may be assumed as a component for determining the selection of the system. For this purpose, the error value should be determined at every point of the investigated sea basin and, based on these values (e.g., by interpolation), the contour line limiting the accuracy areas should be determined, in which the mean positioning error will be in the assumed ranges. The authors will address this area in the next stage of research, aimed at the development of the method presented in this article. To validate the proposed theoretical solutions, a constant mean radar observation error was assumed, which will be discussed further in the article.

On the other hand, as mentioned in the introduction, it is important to ensure a high navigation safety level. In restricted basins, this level may be maintained in different ways. One of the components determining the high safety level is to ensure the appropriate basin depth under the ship keel. In the remainder of this article, the authors will focus on this component as the second criterion in determining the selection of the ship’s self-positioning system.

In the analysis of the needs and requirements to ensure safe and efficient navigation in integrated navigation systems, the decision function will implement the assumed set of undertakings to use a specific positioning system for the self-positioning of the ship. Such a set of undertakings may vary, but it will always depend on the availability of specific positioning systems and safety requirements in the area.

The concept of using a decision function as an a priori object in this article will be limited to the INS analysis of two criteria: (1) the mean positioning error value of one of two available positioning systems; (2) compliance with the safe depth under keel requirement in the restricted basin in the function of the mean ship position error.

The comparative analysis of the mean error representative of the individual positioning systems in the function of the predicted position of the ship in a restricted basin should provide an unambiguous suggestion of the system to use at the given point of the trip. The practical comparative analysis will be reduced to determine if the area of the circle determined by the mean positioning error coincides with the part of the sea basin in which the depth is too small to navigate safely. The analysis enables determining the value of the decision function, which forms Equation (6) for each observation, the given navigation system involved in ship positioning. The function may have the following values: t(y)=0 when the two mentioned areas coincide or t(y)=1 when the areas are separated. When t(y)=0, the analysed positioning system is omitted, and the usability of the next available system is analysed. When t(y)=1, the given system is acceptable to use in the process of ship positioning. Then, one may start forming the a posteriori object and building the decision-robustness matrix defined by Equation (8). Then, finally, one may resolve the estimation task specified by Equation (11).

### 3.3. Attenuation Function as an a Posteriori Object

In navigation practice, one sometimes encounters situations in which measurement results are encumbered with large errors. In general, these errors result from regular mistakes in the identification of marks and the reading of indications of navigation equipment, as well as distortion in data transmission or processing. In most general terms, such errors are referred to as large errors and the observations encumbered with them are referred to as outlying observations. Using such observations in the ship’s self-positioning process may lead to false positioning, with an impact on navigation safety. It seems natural to modify the observation weight as described, e.g., in [37,38] by attenuating the outlying observations. Attenuation is achieved by the determination of the equivalent weight obtained as the product of the original weight and the attenuation function [37,38]:

(14) $${\overset{⌢}{p}}_{i}=t\left(v_{i}\right)p_{i}$$

where ${\overset{⌢}{p}}_{i}$ is the equivalent weight for the i-th observation; $p_{i}$ is the original weight for the i-th observation; $t\left(v_{i}\right)$ is the attenuation function that assumes the value of 1 for standardised corrections $\left(\bar{v}=v/\sigma \right)$ in the acceptable range of $\left(\bar{v}\in \Delta \bar{v}\right)$ or the value from the range of $t\left(v_{i}\right)\in \langle 0,\left.1\right)$, depending on the assumed attenuation function, when the corrections for the completed observations are out of the assumed range of $\Delta \bar{v}=\langle -k,k\rangle$; and $\Delta \bar{v}$ is the acceptable range for random corrections, of which the values are high but still acceptable in the estimation process.

The most used attenuation functions in geodesy are the Huber function, the Hampel function and the Danish function.

The Huber attenuation function [37] has the following form (for $\bar{v}=v/\sigma$ and $\Delta \bar{v}=\langle -k,k\rangle$):

(15) $$t\left(\bar{v}\right)=\left\{\begin{array}{lll} 1 & \mathrm{for} & \bar{v}\in \Delta \bar{v}\\ 0 & \mathrm{for} & \bar{v}\notin \Delta \bar{v} \end{array}\right.$$

hence the following weight function:

(16) $$\overset{⌢}{p}=t\left(\bar{v}\right)p=\left\{\begin{array}{lll} p & \mathrm{for} & \bar{v}\in \Delta \bar{v}\\ 0 & \mathrm{for} & \bar{v}\notin \Delta \bar{v} \end{array}\right.$$

**Figure 1:**
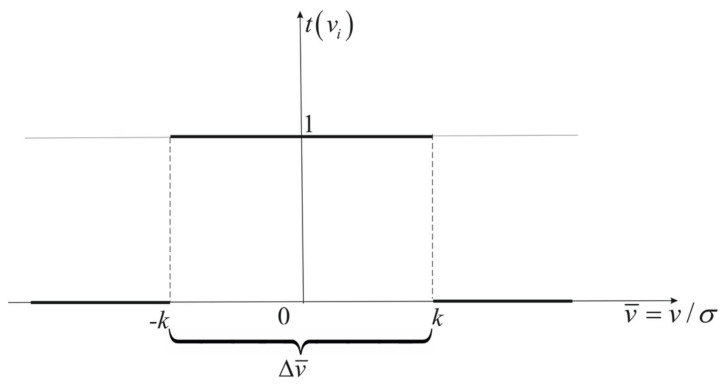
Huber attenuation function.

This function is very radical because it assigns zero values to all weights outside the assumed range, $\Delta \bar{v}$. The Huber function is shown in Figure 1.

In matters related to the navigation process, in which the object is in motion, it is not always feasible to reject or repeat the observation because the object is changing position, and it is almost impossible (and sometimes, due to the time loss, it is pointless) to return to the position in which the observation encumbered with the large error was completed. When the observations available are scarce, it is preferable to deliberately determine one’s position using observations encumbered with errors than not to determine it at all.

**Figure 2:**
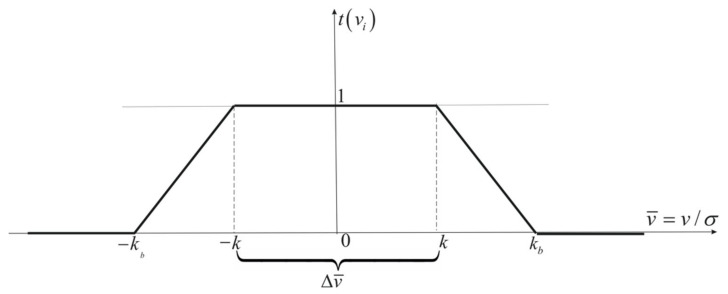
Hampel attenuation function.

Due to the possible decision error margin, the less radical (compared to the above-described) Hampel function is used [38]. In this function, two additional intermediate ranges (Figure 2) are introduced, in which the attenuation function $t\left(v_{i}\right)$ values decrease in a linear manner. The Hampel function takes the following form [38]:

(17) $$t\left(\bar{v}\right)=\left\{\begin{array}{lll} 1 & \mathrm{for} & \bar{v}\in \langle -k,k\rangle \\ \frac{\left|\bar{v}\right|-k_{b}}{k-k_{b}} & \mathrm{for} & \left|\bar{v}\right|\in \left(k,k_{b}\rangle \right.\\ 0 & \mathrm{for} & \left|\bar{v}\right|>k_{b} \end{array}\right.$$

hence the following weight function:

(18) $$\overset{⌢}{p}=t\left(\bar{v}\right)p=\left\{\begin{array}{lll} p & \mathrm{for} & \bar{v}\in \langle -k,k\rangle \\ \left(\frac{\left|\bar{v}\right|-k_{b}}{k-k_{b}}\right)p & \mathrm{for} & \left|\bar{v}\right|\in \left(k,k_{b}\rangle \right.\\ 0 & \mathrm{for} & \left|\bar{v}\right|>k_{b} \end{array}\right.$$

where $k_{b}$ is the limit of additional ranges. The Hampel function is shown in Figure 2.

Although the Hampel function is less radical than the Huber function, it may still “remove” observations encumbered with large errors in the point coordinates determination process. Thus, this function may be useless for navigation as well.

The Danish function has similar properties to the Hampel function, except that, outside the acceptable range of $\Delta \bar{v}$ the function decreases exponentially. Thus, there is no need to distinguish intermediate ranges and their limits. The Danish attenuation function has the following form [39]:

(19) $$t\left(\bar{v}\right)=\left\{\begin{array}{lll} 1 & \mathrm{for} & \bar{v}\in \langle -k,k\rangle \\ \exp\left\{-l{\left(\left|\bar{v}\right|-k\right)}^{g}\right\} & \mathrm{for} & \left|\bar{v}\right|>k \end{array}\right.$$

hence the following weight function:

(20) $$\overset{⌢}{p}=t\left(\bar{v}\right)p=\left\{\begin{array}{lll} p & \mathrm{for} & \bar{v}\in \langle -k,k\rangle \\ \exp\left\{-l{\left(\left|\bar{v}\right|-k\right)}^{g}\right\}p & \mathrm{for} & \left|\bar{v}\right|>k \end{array}\right.$$

**Figure 3:**
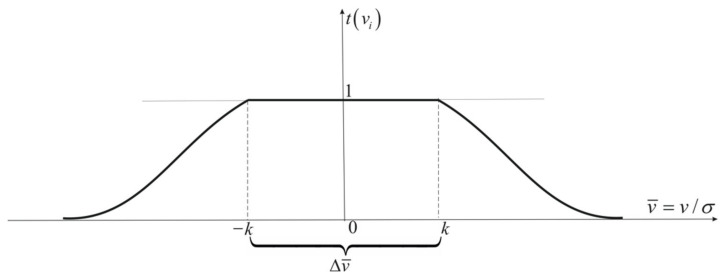
Danish attenuation function.

The values of the attenuation parameters $\left(l,g\right)$ should be selected experimentally. Usually, in the first steps of the iteration process of the estimation task, l=0.01÷0.1 and g=2 are assumed. An improper selection of parameter values causes an unnecessary increase in the number of iteration process steps. The Danish function is shown in Figure 3.

Due to the non-existence of intermediate limits, this function is most useful in the navigation process. The Danish function enables all available observations in the estimation to be used, although they are encumbered with large errors.

## 4. Experimental Results

### 4.1. Computer Application

For the purposes of the validation and visualisation of the proposed method, a computer application with a graphical user interface (GUI) was developed. The application was written in the C# object-oriented language, under NET 4.7.2 framework, in Windows Forms. The application was designed for running in MS Windows 10 or higher. The development environment was MS Visual Studio 2019.

The purpose of this application is to visualise the artificial geographical area with marked radar observations, DGPS observations and positions calculated at different moments and then to execute the decision function and robustness function for the requested inputs. In functional terms, the application may be divided into the following cooperating modules: (1) the a priori object module, including sub-modules: map settings, observation settings and time control, map visualisation and observation and the decision function; (2) the a posteriori object module; (3) the fix positions module.

**Figure 4:**
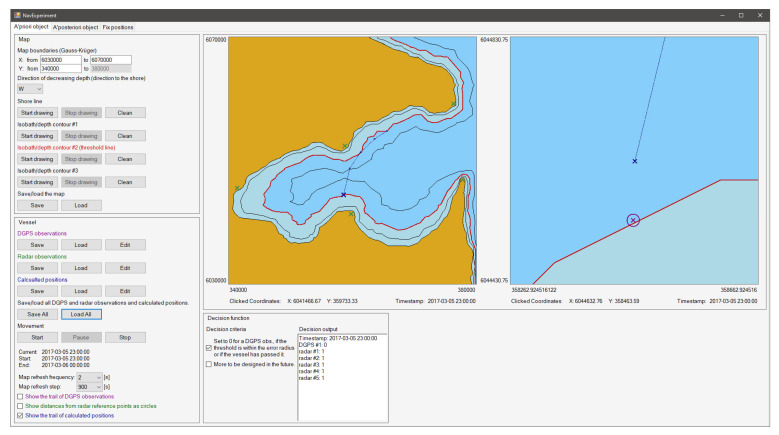
“A priori object” tab in the application for the verification and visualisation of the proposed method.

Figure 4 presents the a priori object module GUI. With regards to map settings, the user of the application may define the limits of the visualised geographical area in Gauss–Krüger rendering and then draw the shape of the coastline and up to three isobaths by moving the cursor over the chart in the central part of the GUI. One of three isobaths will be the threshold for the decision function. The user may save or load map settings to/from the file.

Then, the user should input radar observations, DGPS observations and calculated positions. A single DGPS observation entry consists of the time stamp, geographical coordinates and mean error value (circle radius). A single radar observation entry consists of the time stamp, radar echo distance, radar echo geographical coordinates and mean error. A single calculated position entry consists of a time stamp and geographical coordinates. The user may enter observations manually in accordance with the predetermined application syntax or load them from the file. The observations are visualised automatically on the chart after acknowledgement. The timeline for the entered observations is generated automatically based on time stamps. It enables controlling the passage of time and visualises the motion of the ship. The user may pause, resume, reset and change the time passage rate, as well as the less significant display options.

The central part of the GUI features two charts. The first visualises the entire geographical area, radar echoes, DGPS observation from the current time instant, the position calculated for the current time instant and, optionally, the ship’s route consisting of the positions calculated for all time stamps. The second is a zoom-in view of the first chart, centred on the calculated position, or DGPS observation if the calculated position is not available, for the current instant of the sea trip. From both charts, the user may read the geographical coordinates by clicking on the cursor.

For the decision function, the calculations are made on an ongoing basis for the situation currently shown on the chart. The application presents the decision function values for individual observations. The decision function value for the DGPS observation is 0 if the threshold observation is in the error radius or the observation coordinates exceeded the threshold isobath. If none of the above applies, then the decision function value is 1. It is possible to expand the application to implement more advanced rules for the decision function or use the electronic chart rendering from the electronic chart display and information system (ECDIS) [40].

**Figure 5:**
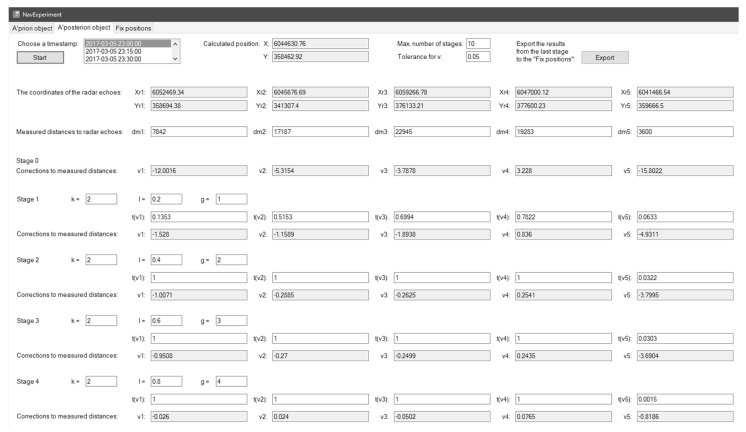
“A posteriori object” tab in the application for the verification and visualisation of the proposed method.

Figure 5 presents the a posteriori object module GUI. This module automatically acquires the required data from the a priori object module. This tab enables the user to perform calculations for robust estimation and determine the corrections to observation for any instant on the timeline independently. The program performs pre-calculations automatically. The user may change the function parameters at any stage independently, and the program will update the calculation results dynamically after each change. In the end, the user may export the results to the fix positions module.

**Figure 6:**
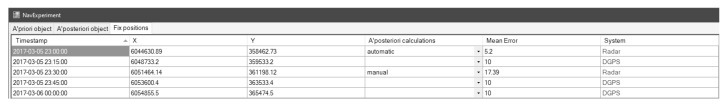
“Fix positions” tab in the application for the verification and visualisation of the proposed method.

Figure 6 presents the fix positions module GUI. The final tab includes the table with the equalised ship position coordinates with mean positioning errors and the navigation system used.

### 4.2. Practical Use of the Decision-Robustness Function in Maritime Navigation

**Figure 7:**
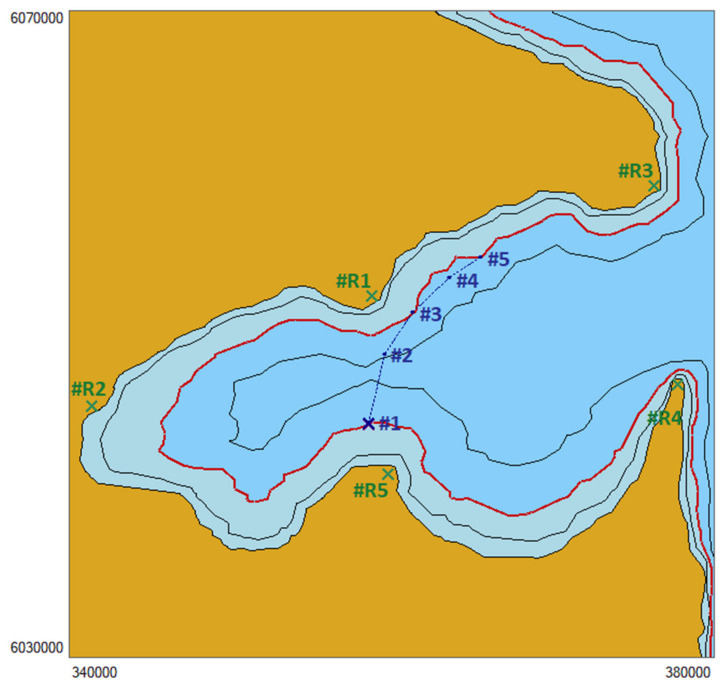
Visualisation of the test basin.

We assumed that the ship moves in a basin, of which the coastline with depth isobaths is shown in Figure 7.

At the following stages of the trip, the ship reaches the calculated positions marked in Figure 7, of which the rectangular Gauss–Krüger coordinates are presented in Table 1.

In each position, the identification of the available navigation systems was completed. In the presented test, the number of available systems was reduced to two (DGPS and radar observations). The following constant fix position error values were assumed: $m_{P_{i}}^{GNSS}=10\;m$ for i=1,…,5 (for the GNSS) and $m_{P_{i}}^{R}=10\;m$ for i=1,…,5 (for radar observations).

**Figure 8:**
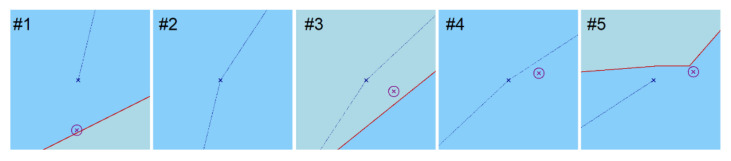
Graphic rendering of the determination of the decision function value (magenta—GNSS, dark blue—calculated position, blue—safe area for navigation, light blue—dangerous area for navigation).

Then, following the assumptions presented above, the analysis was completed to determine the decision function value (the a priori object). The analysis results for the a priori object are shown in Figure 8.

The analysis resulted in the data presented in Table 2.

Considering that the position determined using satellite systems may be more accurate than using radar observation, it was assumed as a rule that in Positions #2, #4 and #5, where t(y)=1, the fix position for further navigation will be the position from the GNSS receiver. In addition, it was considered that the process of building robustness against large errors is included in the algorithms of positioning using satellite measurements, and the coordinates of the fix position of the ship are the final coordinates (Table 3).

However, in Positions #1 and #3, the decision function value for the GNSS is t(y)=0, so the position from the GNSS is not considered in the navigation, but the fix position will be determined using radar observations because the decision function value for this system is t(y)=1. For further calculations in both positions, radar observations were used, of which the coordinates are listed in Table 4.

The radar observations used for further calculations are the measured radar distances (Table 5). Assuming that radar observations may be encumbered with large errors, in the resolution of the estimation task (Equation (11)), the observations of Echo #R5 in Positions #1 and #3 were encumbered with large errors.

#### 4.2.1. Determination of Fix Position #1

In accordance with the robust estimation method, it was first identified whether, and which, observations were encumbered with large errors. To this end, the correction covariance matrix was determined, in (12), and the acceptable range of standardised corrections was specified, $\Delta \bar{\hat{v}}=\langle -2.0;2.0\rangle$. The acceptable range was determined for the trust level γ=95%. The standardised corrections determined according to (13) at the initial (identification) stage were as follows: ${\bar{\hat{v}}}_{1}=-12.0016\notin \Delta \bar{\hat{v}},$ ${\bar{\hat{v}}}_{2}=-5.3154\notin \Delta \bar{\hat{v}},$ ${\bar{\hat{v}}}_{3}=-3.7877\notin \Delta \bar{\hat{v}},$ ${\bar{\hat{v}}}_{4}=3.2280\notin \Delta \bar{\hat{v}},$ ${\bar{\hat{v}}}_{5}=-15.8022\notin \Delta \bar{\hat{v}}$.

All observations were encumbered with larger errors than assumed (the corrections were out of the assumed range). Thus, it was necessary to “process” the observations using the mathematical apparatus described with Equations (9)–(11), re-determine the correction covariance matrix $\left({\hat{C}}_{v}\right)$ and then standardise corrections $\left({\bar{\hat{v}}}_{i}\right)$ for i=1,…,5. The results of the robust estimation procedure may be analysed based on Table 6.

During the subsequent iterations, Echo #R5 was encumbered with large errors (which follows the above-described assumptions). For the analysis, it was assumed that the distance was $d_{\#R5}=3600\;m$; however, the actual distance was $d_{\#R5}=3385.39\;m$. The finally-equalised position coordinates of the ship (Position #1) were:

(21) $${\hat{X}}_{\#1}=X_{\#1}^{0}+{\hat{d}}_{X_{\#1}}=\left[\begin{array}{c} 6,044,630.76\\ 358,462.92 \end{array}\right]+\left[\begin{array}{c} -0.113\\ -0.92 \end{array}\right]=\left[\begin{array}{c} 6,044,630.65\\ 358,462.83 \end{array}\right]$$

with a mean error of $m_{\#1}=0.67\;m$.

#### 4.2.2. Determination of Fix Position #3

Similar to Position #1, it was first identified whether, and which, observations were encumbered with large errors. To this end, the correction covariance matrix was determined $\left({\hat{C}}_{v}\right)$, and the acceptable range of standardised corrections was specified $\left({\bar{\hat{v}}}_{i}\right)$. The acceptable range was also determined for the trust level γ=95%. The standardised corrections determined according to (13) at the initial (identification) stage were as follows: ${\bar{\hat{v}}}_{1}=-6.0593\notin \Delta \bar{\hat{v}},$ ${\bar{\hat{v}}}_{2}=4.2616\notin \Delta \bar{\hat{v}},$ ${\bar{\hat{v}}}_{3}=-7.1601\notin \Delta \bar{\hat{v}},$ ${\bar{\hat{v}}}_{4}=4.0534\notin \Delta \bar{\hat{v}},$ ${\bar{\hat{v}}}_{5}=-11.0565\notin \Delta \bar{\hat{v}}$.

In addition, for Position #3, all observations were encumbered with larger errors than assumed (the corrections were out of the assumed range for standardised corrections). Thus, in this case, the mathematical apparatus can be used to determine the a posteriori object values, re-determine the correction covariance matrix $\left({\hat{C}}_{v}\right)$ and then standardised corrections $\left({\bar{\hat{v}}}_{i}\right)$ for i=1,…,5. The results for Position #3 are listed in Table 7.

During the subsequent iterations, it may be determined that Echo #R5 was encumbered with large errors in this case as well. For the analysis, it was assumed that the distance was $d_{\#R5}=10,300\;m$; however, the actual distance was $d_{\#R5}=10,110.56\;m$. The finally equalised position coordinates of the ship (Position #3) were:

(22) $${\hat{X}}_{\#3}=X_{\#3}^{0}+{\hat{d}}_{X_{\#3}}=\left[\begin{array}{c} 6,051,460.48\\ 361,197.74 \end{array}\right]+\left[\begin{array}{c} 3.664\\ 0.382 \end{array}\right]=\left[\begin{array}{c} 6,051,464.14\\ 361,198.12 \end{array}\right]$$

with a mean error of $m_{\#3}=17.4\;m$.

The application of the decision-robustness function enabled the selection of the appropriate positioning system to determine the fix positions of the ship (Table 8) and using radar observations to detect the ones encumbered with large errors and reduce their impact on the final determinations.

## 5. Conclusions

The diversity and availability of positioning systems in navigation often lead the watchkeeping officer not to analyse which of the available systems would be best used at the given moment but to select the system that enables the easiest determination of the fix position of the ship at sea. At present, satellite systems comprising GNSS are predominantly used. It does not mean, however, that they are the best solution at every stage of the sea trip. Thus, it is worth conducting research aimed at automating the positioning system selection process at every stage of the trip.

The article presents a method of selecting one of the many available (and IMO-compliant [4]) navigation positioning systems. The selection analysis covered the satellite system and the radar system. As can be deduced from the described test, the satellite system, despite its high accuracy, will not always be better than the observations obtained from radar. The reason is that the mean error of determining the ship’s position using the GNSS is always the radius of the circle inside which the position is located. However, it is not possible to determine, without additional analyses, whether this is the actual geographical position of the vessel at sea. However, using radar observations, the ship’s position will always be at sea. If, for a given navigation task, the accuracy of the radar system is sufficient to determine the ship’s position, it is better to use the radar system, not the satellite system, because the position has not been determined on land.

The application of the decision-robustness function in integrated navigation systems is an intelligent tool that may assist not only in the identification and reduction of the impact of observations encumbered with large errors but also in the selection of the appropriate navigation system for self-positioning in restricted basins at any given moment, to ensure the high quality of the determinations.

A popular measure of accuracy in navigation tasks is the mean ship positioning error. This measure enables a comparison of the positioning quality and then the selection of the positioning system, which is part of the INS. It would be useful for the navigator to know the numerical value of this parameter relative to ground positioning systems (e.g., radar-based) at any point in the basin in which the ship moves. A possible visual presentation would be to apply the mean error value contour lines on the sea chart. The authors will address this area in the next stage of research, aimed at the development of the method presented in this article and the acquisition of the fullest possible information on the accuracy measures with an impact on the proper operation of the a priori object described in this article.

Special situational cases (for example, for a radar system: weather, distribution of surrounding reference objects etc.) not described in this paper could be reflected in the parameters of the decision function, which in general could be variable depending on weather conditions. In the presented paper, it has a relatively simple fixed form, but further stages of research could and will focus on more complex decision functions automatically controlled during the voyage. It is conceivable that the parameters of the decision function could change depending on the reading from, for example, the meteorological sensors.

## Figures and Tables

**Table 1:** Coordinates of the ship calculated positions during the test.

Ship’s Positions	X (m)	Y (m)
*#1*	6,044,630.76	358,462.92
*#2*	6,048,890.43	359,497.90
*#3*	6,051,460.48	361,197.74
*#4*	6,053,586.87	363,475.51
*#5*	6,054,839.70	365,398.69

**Table 2:** Decision function values for the individual calculated positions.

Ship’s Positions	GNSS	Radar
*#1*	t(y)=0	t(y)=1
*#2*	t(y)=1	t(y)=1
*#3*	t(y)=0	t(y)=1
*#4*	t(y)=1	t(y)=1
*#5*	t(y)=1	t(y)=1

**Table 3:** Coordinates of the fix position from the GNSS receiver.

Ship’s Positions	X (m)	Y (m)
*#2*	6,048,733.2	359,533.2
*#4*	6,053,600.4	363,533.4
*#5*	6,054,855.5	365,474.5

**Table 4:** Coordinates of radar echoes used in the test.

Radar Echo	X (m)	Y (m)
*#R1*	6,052,469.34	358,694.38
*#R2*	6,045,676.69	341,307.40
*#R3*	6,059,266.78	376,133.21
*#R4*	6,047,000.12	377,600.23
*#R5*	6,041,466.54	359,666.50

**Table 5:** Radar observations used in calculations.

Ship’s Positions	$d_{R}\;(m)$	
*#1*	*#R1*	7842.00
	*#R2*	17,187.00
	*#R3*	22,945.00
	*#R4*	19,283.00
	*#R5*	3600.00
*#3*	*#R1*	2700.00
	*#R2*	20,714.00
	*#R3*	16,852.00
	*#R4*	16,998.00
	*#R5*	10,300.00

**Table 6:** Results of the application of the a posteriori object to radar observations in Position #1.

Step of Iteration	Parameters of the Attenuation Function		Values of the Attenuation Function for Individual Observations					Values of Standardised Corrections for Individual Observations				
	l	g	$t({\bar{v}}_{1})$	$t({\bar{v}}_{2})$	$t({\bar{v}}_{3})$	$t({\bar{v}}_{4})$	$t({\bar{v}}_{5})$	$\left|{\bar{v}}_{1}\right|$	$\left|{\bar{v}}_{2}\right|$	$\left|{\bar{v}}_{3}\right|$	$\left|{\bar{v}}_{4}\right|$	$\left|{\bar{v}}_{5}\right|$
1	0.2	1	0.135	0.515	0.699	0.782	0.063	1.528	1.158	1.893	0.835	**4.931**
2	0.4	2	1	1	1	1	0.032	1.007	0.289	0.263	0.254	**3.800**
3	0.6	3	1	1	1	1	0.03	0.951	0.270	0.250	0.244	**3.690**
4	0.8	5	1	1	1	1	0.00002	0.023	0.039	0.040	0.068	0.086

**Table 7:** Results of the application of the a posteriori object to radar observations in Position #3.

Step of Iteration	Parameters of the Attenuation Function		Values of the Attenuation Function for Individual Observations					Values of Standardised Corrections for Individual Observations				
	l	g	$t({\bar{v}}_{1})$	$t({\bar{v}}_{2})$	$t({\bar{v}}_{3})$	$t({\bar{v}}_{4})$	$t({\bar{v}}_{5})$	$\left|{\bar{v}}_{1}\right|$	$\left|{\bar{v}}_{2}\right|$	$\left|{\bar{v}}_{3}\right|$	$\left|{\bar{v}}_{4}\right|$	$\left|{\bar{v}}_{5}\right|$
1	0.2	1	0.444	0.636	0.356	0.663	0.163	**2.356**	**2.505**	**2.717**	**2.041**	**5.915**
2	0.2	2	0.975	0.950	0.902	1	0.047	0.945	0.663	1.094	0.508	**3.915**
3	0.6	3	1	1	1	1	0.015	0.367	0.246	0.334	0.137	**2.277**
4	4.5	0.005	1	1	1	1	0.011	0.301	0.203	0.246	0.096	2.008

**Table 8:** Results of the application of the a posteriori object to radar observations in Position #3.

Ship’s Positions	X (m)	Y (m)
*#1*	6,044,630.6	358,462.8
*#2*	6,048,733.2	359,533.2
*#3*	6,051,464.1	361,198.1
*#4*	6,053,600.4	363,533.4
*#5*	6,054,855.5	365,474.5

## Data Availability

Not applicable.
